# Food-Origin Lactic Acid Bacteria May Exhibit Probiotic Properties: Review

**DOI:** 10.1155/2018/5063185

**Published:** 2018-10-01

**Authors:** Dorota Zielińska, Danuta Kolożyn-Krajewska

**Affiliations:** Department of Food Gastronomy and Food Hygiene, Warsaw University of Life Sciences (SGGW), Nowoursynowska 159, 02-776 Warsaw, Poland

## Abstract

One of the most promising areas of development in the human nutritional field over the last two decades has been the use of probiotics and recognition of their role in human health and disease. Lactic acid-producing bacteria are the most commonly used probiotics in foods. It is well known that probiotics have a number of beneficial health effects in humans and animals. They play an important role in the protection of the host against harmful microorganisms and also strengthen the immune system. Some probiotics have also been found to improve feed digestibility and reduce metabolic disorders. They must be safe, acid and bile tolerant, and able to adhere and colonize the intestinal tract. The means by which probiotic bacteria elicit their health effects are not understood fully, but may include competitive exclusion of enteric pathogens, neutralization of dietary carcinogens, production of antimicrobial metabolites, and modulation of mucosal and systemic immune function. So far, lactic acid bacteria isolated only from the human gastrointestinal tract are recommended by the Food and Agriculture Organization (FAO) and World Health Organization (WHO) for use as probiotics by humans. However, more and more studies suggest that strains considered to be probiotics could be isolated from fermented products of animal origin, as well as from non-dairy fermented products. Traditional fermented products are a rich source of microorganisms, some of which may exhibit probiotic properties. They conform to the FAO/WHO recommendation, with one exception; they have not been isolated from human gastrointestinal tract. In light of extensive new scientific evidence, should the possibility of changing the current FAO/WHO requirements for the definition of probiotic bacteria be considered?

## 1. Introduction

The role of food in developing human health and wellbeing has been known since the times of Hippocrates, whose saying, “Let food be thy medicine and medicine be thy food,” frequently repeated today, has become the slogan of supporters of “treating” with food. This correlation is particularly apparent and documented as regards the beneficial microflora found in the human body.

An efficiently working gut ecosystem, the so-called microbiome (quantitative and qualitative composition of various microorganisms) has a great impact on the person's ability to maintain their health. The microflora in human intestines is the most varied ecosystem on earth in terms of species (100–1000 species). The microbiome influences many physiological systems, including immunity or mental state. Due to the growing awareness of the role that the gut microflora has on people keeping their health, for over 20 years research work has been conducted worldwide, with regard to the possibilities of modifying positively or enriching human microbiome. This is because it has been noticed that more and more both quantitative and qualitative composition of the gut microflora diverges from the norm. These changes are caused by many endogenous factors (connected directly with the person, i.e., viral or bacterial infections) and exogenous (foodstuffs, steroids, laxatives, antibiotics and chemotherapeutics, contraceptives, etc.), which directly result in numerous disorders connected not just with the human digestive system. It is believed that the use of appropriately selected strains of probiotic bacteria in nutrition may result in beneficial modulation of the composition of the gut bacterial flora [1].

Probiotics are an important concept for health care in the 21st century. The global probiotics market size was valued at USD 35.9 billion in 2016. In Asia and Europe, probiotics are widely used as health foods and medicines. In the global probiotic market, the European market is the largest and the fastest growing with an average annual growth rate of around 20% [2]. Development of efficient strains of probiotics is a key industry scenario. The health benefits of probiotics and rising awareness among the consumers are expected to drive the industry growth over the next few years. The global revenue generated from probiotics market is estimated to be valued at roughly US$ 6,762.2 million by the end of 2018 and is expected to increase in the near future [3].

The main purpose of the review was to discuss the current definition of probiotic and summarize current understanding of probiotic in the view of the use of nonhuman isolation sources. Additionally, we conduct a comparative review of the latest literature investigating candidates to probiotic strains isolated from different sources to identify their common features.

## 2. Definitions and Legal Regulations concerning Probiotics

The definition of probiotics changes together with the development of knowledge about them. A definition of the probiotic was proposed in 2001 by Schrezenmeir and De Vrese: “a preparation of or a product containing viable, defined microorganisms in sufficient numbers, which alter the microflora by implantation or colonization, in a compartment of the host and by that, exert beneficial effects on host health” [1].

In 2002, FAO and WHO experts adopted a definition of probiotics deciding that these are “live microorganisms which when administered in adequate amounts confer a health benefit on the host.” Microorganisms, in order to be classified as probiotic strains, should be defined precisely by determining appropriate criteria concerning safety of their use and functional and technological features. Microorganisms, candidates for the name “probiotics,” must meet three key requirements [4]:

1. They must be living at the moment of administration and must be microorganisms
2. They must be administered in a dose which is sufficiently high to have a health promoting effect. The recommended effective dose is strictly connected with the clinical documentation on which it must be based
3. Microorganisms administered must have a beneficial effect on the host

Probiotics must be identified at the strain level and safe for use in humans. They must have a beneficial effect on the host; this is why they should originate from the gastrointestinal tract of a healthy individual and be resistant to gastric enzymes, low pH, and high concentration of bile salts [5].

However, it is currently believed that it is the specific way in which they work and not the source of isolation of the microorganism that is important. Most of probiotic strains used in humans have been isolated from people; however this recommendation does not constitute a requirement. There are some well-tested probiotic strains known that do not originate from human hosts (e.g.,* Bifidobacterium animalis* subsp.* lactis* and* Saccharomyces cerevisiae* var.* boulardii*). David et al. [6] showed that several microbes found in consumed food products such as cheese and deli meats were reisolated from faecal samples of individuals who consumed them. Microorganisms of food-ingested bacteria made up more than 1% of the faecal microbiome in some cases. In reality, it is very difficult to confirm the source of origin of a microorganism [4]. This is why it is believed that probiotics intended for people require proving that they work in human hosts. It is also recommended that strains isolated from a population in which they are to be later applied are used in probiotic preparations [7]. Procedures which are to confirm health properties of the tested probiotic strains have been developed by FAO/WHO [8].

In 2014, experts from ISAPP (International Scientific Association for Probiotics and Prebiotics) organised a meeting which resulted in a publication verifying the previous Report of FAO/WHO [8]. A consensus was announced which allowed for minor grammatical corrections to the definition, retaining its sense and meaning. The term “probiotic” may be used to refer to many types of microorganisms which demonstrate health benefits for the host, while remaining alive. In the document presented, this feature was emphasised particularly, and metabolites as well as dead cells of microorganisms were excluded from the definition of a “probiotic.” Additionally, it was agreed that “probiotics” are not undefined consortia of microorganisms (such as faecal microbiota transplants) or fermented foods containing undefined microorganisms (Table 1) [9].

The reason for undertaking the discussion was the fact that both scientific research and clinical evidence progress fast, similar to the development of a number of probiotic products. Unfortunately, the incorrect use of the term “probiotic” has become a serious problem because in the case of many products this term is used while the required criteria are not met. At the same time, probiotic products came to the justified attention of regulatory bodies protecting consumers from misleading health claims.

In recent years, a new concept appeared, suggesting that key health benefits connected with probiotic mechanisms may be assigned to the species and not just to specific strains of microorganisms, in particular in the case of some species of lactic fermentation bacteria, the strains of which have been used as probiotics for a long time. It is believed that if fermented food (e.g., sauerkraut) contains a large number of live cells belonging to the species for which health benefits have been proven (e.g.,* Lb. plantarum*), it may be reasonably supposed that this food may be deemed showing similar health benefits to the benefits arising from the probiotic bacteria of the same species [10].

Scientists and ISAPP experts have emphasised the importance of a lot of evidence showing the connection between eating fermented foods and human health, such as reduction of the risk of diabetes type 2 development in people eating fermented milk products. However, difficulties arise as regards unequivocal indication of the participation and role of live microorganisms in those mechanisms. Health-promoting microorganisms found in fermented foods often constitute consortia undefined at the strain level. It is recommended that this type of food is described only as “containing live and active microorganism cultures” [9].

The term “postbiotics” has also arisen. This term is used to determine nonviable bacterial products or metabolic byproducts produced by probiotic microorganisms which show biological activity in the host. Generally, postbiotics include bacterial metabolites, byproducts, such as bacteriocins, organic acids, ethanol, diacetyl, acetaldehydes, and hydrogen peroxide. It has been found, however, that also some heat inactivated probiotics may retain important cellular structures and exert biological activity in the host's body [1].

Some research has shown that dead cells of probiotic bacteria may have beneficial effects, such as modulation of the immune system and binding carcinogens in the host's body; however their effect is weaker or restricted. It is suggested, moreover, that the application of inactivated cells is a solution better from the point of view of safety of use, particularly in the case of newborn babies or patients with immunosuppression [11]. It is therefore sufficient for probiotic strains to increase their number accordingly at the initial stage of production, until the required number of microorganisms is obtained in the product, whereas later, during the storage process, they do not have to display good viability [1]. Taking this into account, we propose extending the framework for probiotic products to include the concept of postbiotics and inactivated bacterial cells (Table 1).

The issue of defining what probiotics are and what they are not is important for many groups, including consumers, scientists, health care professionals, industry representatives, and legislators. Also the determination of which food products may be deemed probiotic, due to the potentially important interventions to improve health and wellbeing, requires further classification. The approach to these issues, however, is not identical all over the world.

In most countries only very general health claims are currently permitted to label foods containing probiotics. A FAO/WHO taskforce [8] recommended specific health claims, permitted for food products with probiotics if sufficient scientific evidence is available. It is recommended that it is the producer who is responsible for the product, but an independent third party should check whether health claims are true and are not misleading.

In the EU, food containing probiotic bacteria is subject to general community law regulations. In accordance with the suggestion of FAO/WHO [8], in 2006, the European Parliament and the Council published Regulation No. 1924/2006 on “Nutrition and Health Claims Made on Foods” [12]. The Regulation concerns all nutrition and health claims for all types of foods intended for end consumers, including probiotic products marketed with health claims. The purpose of the Regulation is to harmonise health claims on the European level in order to protect consumers better, including in retail communication (labelling, presentation, and promotional campaigns) as well as trademarks and brands which may be interpreted as nutrition or health claims. The Regulation also establishes authorisation procedures required to ensure that claims on labels, in presentations and food advertisements, are clear, concise, and based on scientific evidence. The European Food Safety Authority (EFSA) developed a scientific and technical guide for application in order to obtain consent for the use of health claims.

EFSA may express its consent to the placing of health claims on health product packaging on the basis of scientific evidence collected. However, so far EFSA has not expressed its consent to placing claims concerning probiotics on any of the products available on the EU market, thus blocking the development of probiotic foods [13]. The only EU country which published a list of probiotics and a guide concerning their use is Italy [14].

The organisation ESPGHAN (the European Society for Paediatric Gastroenterology, Hepatology, and Nutrition) operating under the EU aegis in 2014 published strong recommendations for the clinical use of two probiotics (*Lactobacillus rhamnosus* GG and* Saccharomyces boulardii*), stating the dosage in the case of children treated for acute diarrhoea, acute gastroenteritis, and postantibiotic diarrhoea. At the same time, the document emphasises the low quality of evidence for those recommendations, and the list of microorganisms which may not be used due to the very low quality of evidence was provided, and a microorganism was indicated (*Enterococcus faecium* SF68), the use of which is not recommended for safety reasons [15].

Nutritional recommendations vary depending on the country because the consumption of nutrients and the priority in selecting main nutrients may depend on the availability of foods and nutritional preferences. In EU Member States, main food groups under national nutrition guidelines do not vary significantly, but there are differences in types of foods in groups and quantities recommended for consumption. At the moment, there are no harmonised guidelines at the EU level due to the lack of representative data concerning consumption. In Europe, for example, most of the national nutrition recommendations include yoghurt as part of a healthy diet. Similar recommendations are also given in non-European countries, e.g., New Zealand, USA, and Australia [16].

Health Canada decided that generally positive impact on health may be expected, without evidence as to the strain for the following microorganisms stated in the quantity of 10^9^ units/dose:* Bifidobacterium (adolescentis, casei, fermeanimalis, bifidum, breve,* and* longum) *and* Lactobacillus (acidophilus, casei, fermentum, gasseri, johnsonii, paracasei, plantarum, rhamnosus,* and* salivarius*). It was deemed that this is a group of well-investigated species, having general health benefits, particularly for the digestive system and immune system; therefore in Canada a general claim is used: “promotes a healthy gut flora” [17, 18].

The law is different in the USA where a lot of microorganisms listed in the document entitled Clinical Guide to Probiotic Products [19], with recommendations for use and dosage, are permitted.

Many medical organisations have evaluated probiotics and probiotic foods in terms of their proven health benefits. Such evaluations resulted in clinical recommendations of medical organisations concerning the use of selected well-tested probiotics in specific clinical conditions, such as the treatment and prevention of acute gastroenteritis, necrotising enterocolitis, and postantibiotic diarrhoea, as well as in the supplementation of milk for initial newborn nutrition [16]. Markowiak and Śliżewska [7] in their review presented an extensive list of possible clinical uses of probiotics in the case of various conditions.

## 3. New Sources and Types of Probiotics

The conventional source of probiotics for human use, recommended by FAO/WHO, is the human gastrointestinal tract (GIT). The number of microorganisms inhabiting the GIT has been estimated to exceed 10^14^, most of them belonging to the domain Bacteria. Compiled data from the Human Microbiome Project studies identified 2172 species isolated from human beings, classified into 12 different phyla. Around 90% of all the bacterial taxa belong to just two divisions: Bacteroidetes and Firmicutes. The other divisions that have been consistently found in samples from the human distal gut are Proteobacteria, Actinobacteria, Fusobacteria, and Verrucomicrobia. Only very few species of Archaea (mostly* Methanobrevibacter smithii*) seem to be represented in the human distal gut microbiota. Eukaryotes (yeasts and protists) and Viruses (phagi and animal viruses) are also present [20, 21].

Many of the probiotic strains have been isolated from human intestine, such as* Lb. salivarius *subsp.* salicinius* and* Lb. acidophilus* [22], as well as from human faeces, such as* B. longum* and* Lb. acidophilus*, and less frequently from human stomach such as* Lb. fermentum*,* Lb. gasseri*,* Lb. vaginalis*,* Lb. reuteri*, and* Lb. salivarius *[23].

A common conception is that probiotics, upon consumption, must withstand gastrointestinal transit and always colonize the intestines for benefits to be observed [24]. In fact, certain probiotics (e.g.,* B. longum *and* Bacteroides thetaiotaomicron*) colonize the human intestinal microbiota, but others (e.g.,* Lb. casei* and* B. animalis*) do not. For the health benefits of probiotics, there is no proof of a need for colonization, and mostly probiotics reside only transiently following food intake [25]. It has been claimed that probiotics isolated from human and animal intestines have different adhesion capacity than probiotics originating from food and other unconventional sources. Intestinal isolates usually exhibit higher adhesion activity than the food-origin isolates [26]. However, Monteagudo-Mera et al. [27] reported that some* Lactobacillus* strains isolated from cheese were more adherent to CaCo-2 cells than* Lactobacillus* spp. isolated from human faeces.

It is also worth noting that commensals in the gut can be the source of probiotic strains, but until these strains are isolated, and carefully characterized for their health effects, they cannot be called “probiotics.” The distinction between commensal microorganisms and probiotics was highlighted by an ISAPP panel [9].

Several probiotic bacteria of human origin are used commercially, like* Lactobacillus rhamnosus* GG,* Lactobacillus casei* Shirota, and* Lactobacillus acidophilus *LA-1. However, several commercially explored, well-studied probiotic strains are species that are not native human colonizers (e.g.,* Bifidobacterium animalis* subsp.* lactis* and* Saccharomyces cerevisiae* var.* boulardii*) [4, 28].

Probiotic microorganisms, beside the conventional source (a healthy person's gastrointestinal tract), may originate from unconventional sources, such as the gastrointestinal tract of an animal, human breast milk, food (fermented and unfermented), air, or soil. In Table 2 there are examples of conventional and unconventional sources of probiotics isolation only from the recent years. Looking for probiotic properties of food-origin lactic acid bacteria becomes a visible trend in food microbiology researches. When the level of advancement of the researches on probiotic candidates (in most cases only* in vitro* tests) is analysed, it becomes clear that the investigations are still at the beginning of a long way. Even more so, the correlation of* in vitro* with* in vivo* results remains obscure [29]. However, abundance of the current findings seems to be extremely promising.

It has been recommended that microorganisms used for production of probiotic animal formulas should be isolated from individuals belonging to the species for which they are intended, because part of health beneficial effects is probably species specific [81]. Therefore the guts of several animal species are good sources of probiotics, mainly for animal use. Billet et al. [82] have found that* Bifidobacterium actinocoloniiforme *R-53049, isolated from bumblebee gut, showed the potential to colonize the bumblebees' guts permanently after administration. Recently, three candidate probiotic strains,* Bacillus subtilis* (IPA-S.51) and* Shewanella algae* (IPA-S.252 and IPA-S.111) isolated from shrimp found to be active* in vivo* against the pathogenic bacteria* Vibrio sp.,* improved shrimp growth and could develop in shrimp hepatopancreas and intestine [83]. Literature shows many other examples of probiotics isolated from animal intestinal tracts for animal use, such as swine [84], poultry [85], marine, and freshwater fish [86].

Among the lactic acid bacteria isolated from** breast milk** three species clearly predominated:* Lb. gasseri*,* Lb. reuteri*, and* E. faecium*. These species are considered among the probiotic bacteria. Recently Rajoka et al. [87] isolated* Lactobacillus* sp. from mother's milk and examined them for resistance to acid and bile and antioxidant properties as well as antibiotic susceptibility. Moreover, they have found that tested* Lactobacillus *strains were efficient against cervix cancer cells and hold promise to show probiotic features. Arroyo et al. [88] investigated the efficacy of* Lb. fermentum* CECT 5716 or* Lb. salivarius *CECT 5713 isolated from breast milk, to treat lactational mastitis. They found that probiotic treatment led to a significant reduction in the milk bacterial count and to a rapid improvement of woman condition. Other authors also claimed that lactic acid bacteria that are originally isolated from human milk may have an endogenous origin and may not be the result of contamination from the surrounding breast skin and therefore would fulfil some of the main criteria generally recommended for human probiotics [89, 90].

**Milk of farm animals **and milk products constitute a good source of the lactic acid fermentation bacteria. Spontaneously fermented milk products are still produced until this day in many parts of the world and constitute an excellent source of probiotic microorganisms, particularly bacteria from the genera* Lactobacillus, Lactococcus*, or* Streptococcus, *as well as yeasts. The examples include drinks,* kule naoto*, Masai fermented milk [91] and Koumiss [92], from which microorganisms with immunomodulatory properties have been isolated. Unconventional sources of isolation of microorganisms with probiotic characteristics were also yak milk [93] and camel milk [94] and goat milk [95] as well as other fermented milk drinks. For example,* Lactobacillus kefiranofaciens* XL10, with a high yield of extracellular polysaccharide (EPS), isolated from Tibetan kefir grain has been considered to exhibit probiotic potential* in vitro* and* in vivo* [56]. Recently Bengoa et al. [96] evaluated the adhesion ability* in vitro *of* Lb. paracasei* strains isolated from kefir grains after acid and bile stress and observed that, after gastrointestinal passage,* Lb. paracasei* strains have increased their ability to adhere to mucin and epithelial cells.

Many** regional cheeses **in Europe have also been used to isolate microorganisms with health promoting properties [54, 97, 98]. Grigoryan et al. [99] demonstrated that* Lactobacillus helveticus* INRA-2010-H11 isolated from the Chanakh cheese from Armenia exhibited a high aggregation and adhesion activity* in vitro* and* in vivo*, so it has the potential as a good probiotic strain. Recently, also four* Enterococcus* strains isolated from a regional Argentinean cheese were found to be safe, and authors promoted these strains for further study and suggest their utilization as adjuvant in a starter culture for cheese production [100].

Microorganisms (bacteria and fungi) with probiotic properties are also isolated from other fermented and unfermented products of animal origin, such as** meat and raw cured cold meats **[101, 102],** fish and seafood **[62, 103, 104], or** honey **[105].

Han et al. [59] evaluated* in vitro* probiotic properties of four strains* Pediococcus pentosaceus *R1,* Lactobacillus brevis *R4,* Lactobacillus curvatus* R5, and* Lactobacillus fermentum* R6 isolated from Harbin dry sausages. They found that these strains tolerated the human gastrointestinal (GI) tract well and possess antioxidant activity. Recently, also Hernández-Alcántara [2018] isolated six thermotolerant lactic acid bacteria from cooked meat products and showed that* E. Faecium *UAM1 has probiotic properties that predict its capability to colonize in competition with pathogens in the intestinal tract.

Recently Yamada et al. [63] have found that heat-killed* Lb. paracasei *NFRI 7415 isolated from traditional Japanese fermented fish (funa-sushi) possess* in vitro* probiotic characteristics and inhibited mesangial proliferative glomerulonephritis by alcohol intake with stress in mice model.

Hamdy et al. [106] investigated* Bacillus subtilis *HMNig-2 and* Bacillus subtilis* MENO2 isolated from honey and bee gut and found that these strains and prebiotic levan exhibited* in vivo *promising probiotic characteristics, such as immune system improvement and protection from* Salmonella typhimurium* infection and their associated effects on liver such as inflammation and hepatic infiltration.

It has been shown that** fruit, vegetables, juices, and grain products **are an equally valuable source of isolation [107–109].

Recently,* Lactobacillus fermentum* TcUESC01 (LF) and* Lactobacillus plantarum* TcUESC02 (LP) isolated from the fermentation of cocoa (*Theobroma cacao* L.) were evaluated* in vitro* and* in vivo* as probiotics. The protective effect of administration of the lactobacilli against* Salmonella typhimurium* was proved [68].

Other authors isolated 150 yeasts from peel and spontaneously fermented pineapple pulp. Five of them survived the gastrointestinal conditions and showed antibiotic resistance and autoaggregation properties, which predisposes them for further probiotic characteristics study [74].

Also other unconventional sources, such as** soil **[80],** air **from rooms in which the leavening for the production of sourdough bread has been prepared [79], or** sewage, kitchen leftovers, and postproduction waste **[77, 110], have become a source of isolation of bacteria with probiotic properties.

**Also in our laboratory**, in the Department of Technology Catering and Food Hygiene at Warsaw University of Life Sciences, the investigations were performed on isolation, identification, and characterization of lactic acid bacteria, mainly* Lactobacillus,* and the characteristic of probiotic and functional properties of these strains. Currently, the collection possesses over 200 pure cultures of* Lactobacillus* sp. and other lactic acid bacteria isolated from spontaneously fermented food products.

Traditional and regional Polish fermented food products were found as an abundant source of potential probiotic strains. For example, twenty-one strains of the genus* Lactobacillus* and the genus* Pediococcus*, isolated from raw fermented meat products, were found to be resistant to gastric enzymes, low pH, intestinal enzymes, and bile salts. Moreover few strains had the ability to produce bacteriocins or bacteriocin-like substances. Most strains were considered as safe. In conclusion, strains* Lb. brevis* SCH6 and* Pd. pentosaceus* BAL6 and KL14 were selected as potential probiotic, as well as a viable bioprotective culture that can be inoculated in raw fermented meat products as starter cultures [60].

In other investigations of the same authors, twenty-five strains were isolated from raw, organic whey samples, and sixteen of them were identified as* Lb. plantarum* and* Lb. fermentum. *The study showed that all of the strains had *β*-galactosidase activity and average lipolytic, esterolytic, and low proteolytic activity. Some of them reduced nitrate content. Moreover most of the tested strains were susceptible to known antibiotics and few of* Lb. plantarum* and* Lb. fermentum* strains did not possess any transfer resistance genes. The study reveals that the* Lactobacillus *strains isolated from organic whey are safe and have high potential for food application. Moreover these strains were highly active against selected pathogens, such as* E. coli, L. monocytogenes, Salmonella enteritidis*, and* Shigella* sp. [53].

In the study of Ołdak et al. [55] 29 of* Lactobacillus plantarum* strains isolated from Oscypek and Korycinski, the traditional and regional cheeses form Poland, were investigated. It has been found that the highest antimicrobial activity was observed for* L. monocytogenes*; however, the level of that activity was different depending on the* Lb. plantarum* strain. Moreover, the antagonistic activity shown by* Lb. plantarum* strains was connected with the source from which a given strain was isolated. Strains isolated from the Oscypek cheese represented stronger activity against* L. monocytogenes*, whereas strains isolated from the Korycinski cheese were more active against* E. coli*.

Abundant sources of* Lactobacillus* strains were also found in Polish food product of plant origin, such as traditional fermented cabbage and cucumber. Zielińska et al. [67] isolated 38 strains from the pickled samples and 14 were identified as* Lactobacillus* spp. The study showed that all tested strains were resistant to harmful gastrointestinal conditions (pH 2.5, 0.2% bile salt solution, and 0.4% phenol addition); however pH 1.5 caused death of* Lactobacillus* cells, except 4 strains, which could survive for 90 min at pH 1.5. The hydrophobic nature of the cell surface of the tested strains suggested their adhesion capacity. On the basis of the results, 10 of the selected* Lactobacillus *strains are considered safe and can survive under gastrointestinal conditions, which requires them to undergo future* in vitro* and* in vivo* studies. In the next study [111], the selected strains were screened for adhesion capacity to Caco-2 cells, regulation of selected cytokine production by incubating bacterial suspensions with THP-1 macrophage like cells, and stimulation of Caco-2 cells apoptosis using a Capase-3 assay. The results of the research work carried out so far have been presented at conferences and partially published.

Based on our results, we can conclude that the properties investigated (antagonistic, enzymatic activity, susceptibility, or resistance to selected antibiotic) of tested lactic acid bacteria strains were dependent on the source of isolation. For example, strains isolated from the Oscypek and Korycinski cheeses and fermented vegetables were more active against* Listeria *than strains isolated form fermented meat and whey. The weakest activity of the strains tested occurred against* E. coli* and* Salmonella*; however strains isolated from the Korycinski cheese and strains isolated from fermented cucumber were found to be moderately active against those pathogens. On the other hand, strains isolated from organic whey were more susceptible to selected antibiotics than strains isolated from other sources.

## 4. Summary

Despite the widely conducted research and extensive scientific evidence, there are still no clear-cut legal requirements, which leads to inappropriate application, or even abuse of the term “probiotic.” In accordance with the current state of knowledge, probiotic organisms should show an effect of improved health in the host's body. The origin of the microorganisms from the human gastrointestinal tract is not a criterion that is indicated as essential. The more so as more and more scientific evidence indicates new unconventional sources of isolation as correct ones.

Isolation, identification, and assessment of safety and probiotic properties of new, “wild” strains of microorganisms from traditional foods constitute a necessary practice, particularly in order to develop the technology of production of food-dedicated vaccines. New vaccines, besides protective properties (bacteriostatic and bactericidal), may provide additional values connected with the consumer's improved health. Microorganisms isolated from foods show better viability in the food environment and guarantee more attractive sensory characteristics in comparison with microorganisms originating from intestines [112]. The most frequently encountered types of probiotics are* Bifidobacterium* (*adolescentis, animalis, bifidum, breve*, and* longum*) and* Lactobacillus* (*acidophilus, casei, fermentum, gasseri, johnsonii, paracasei, plantarum, rhamnosus*, and* salivarius*). Selected strains of yeasts are also believed to be probiotic:* Saccharomyces boulardii*. The* Escherichia coli* or* Bacillus coagulans* strains are used less frequently. Recently, interest in newly identified human commensals has been growing:* Akkermansia muciniphila, Faecalibacterium prausnitzii, Roseburia *spp., and* Eubacterium hallii, *which are referred to as “probiotics of the future.” Thanks to new possibilities of growing these bacteria, which due to their properties (strict anaerobes) were believed as noncultivated, the interest of researchers and possibilities for identifying their phenotype increased. In the future, there are plans to use the newly found strains to design ecosystems which may be used to replace the microbiome in people with various conditions for therapeutic purposes. The possibilities of using those microorganisms in the production of food will depend on the progress in further research and proving the safety of their application in human beings [9].

Evidence from well-conducted observation research and a lot of randomised controlled research confirms the potential influence of probiotics on human health. However, extending the term “probiotic” to include bacteria isolated from traditionally, spontaneously fermented foods seems justified. Microorganisms isolated from fermented products constitute the microflora of an environment in which the products were produced. If they are tested, particularly in terms of their probiotic properties and safety, they may constitute an interesting alternative to gut bacteria.

## Figures and Tables

**Table 1:** Overall framework for probiotic and nonprobiotic products.

Live cultures		Not-live cultures
Probiotics	Not-probiotics	
(1) Probiotic drugs (2) Probiotic medical foods (3) Probiotic foods (4) Non-oral probiotics (5) Probiotic animal feed (6) Defined microbial consortia (7) Probiotic dietary supplement (8) Probiotic infant formula	(1) Fermented foods with undefined microbial content (2) Undefined consortia including faecal microbiota transplant	(1) Postbiotics (2) Inactivated bacterial cells

According to [1, 9].

**Table 2:** Examples of conventional and unconventional sources of probiotic microorganisms.

Source of isolation		Strains identify	Activities	References
Human	**Gastrointestinal tract**			
	(i) stomach	10 of *Lb. gasseri*, *Lb. fermentum*, *Lb. vaginalis*, *Lb. reuteri *and *Lb. salivarius strains*	*In vitro* gastrointestinal conditions resistance, antimicrobial activity	[23]
		2 of *Lb. reuteri* strains among 19 isolates	*In vitro* gastrointestinal conditions resistance, antimicrobial activity, adhesion to epithelial gastric cell line, antioxidative activity antibiotic resistance	[30]
	(ii) intestine	*Lb. rhamnosus* IMC 501 and *Lb. paracasei *IMC 502,* Lb. plantarum *319	*In vivo* gastrointestinal conditions resistance, adhesion to HT-29 cells, antimicrobial activities, antibiotic susceptibility and plasmid profile. *In vivo* survival through intestine in a 3 months human feeding trial	[31]
		*Lb. rhamnosus* IMC 501 and *Lb. paracasei* IMC 502	*In vivo *improvement of intestinal microbiota with beneficial microbes and enhances bowel habits of healthy adults.	[32]
		*Lb. helveticus* BGRA43	*In vitro *gastrointestinal conditions resistance, adhesion to Caco-2 cells, antimicrobial and proteolytic activity	[33]
		*Lb. fermentum* BGHI14 and *Lb. helveticus* BGRA43	*In vitro *antimicrobial effect on *C. difficile, *immunomodulatory activity, increase proliferation of GALT lymphocytes *In vivo* reduction of *C. perfringens* in goats	[34]
	(iii) feaces	10 of *Faecalibacterium prausnitzii* strains	*In vitro* adhesion to HT-29 cells, antimicrobial and antibiotic activity, immunomodulatory properties, Short Chain Fatty Acid production	[35]
		*Lb. casei*/*paracasei *CTC1677, *Lb. casei*/*paracasei *CTC1678 and *Lb. rhamnosus *CTC1679	*In vitro* gastrointestinal conditions resistance, antimicrobial and antibiotic activity, auto-aggregation *In vivo *survival, colonize and persist in the gastrointestinal tract in a human intervention study	[36, 37]
		*Lb. fermentum* F53 and KC5b,* E. gallinarum*, and *E. faecalis* strains	*In vitro* gastrointestinal conditions resistance, assimilation of cholesterol	[38]
	**Breast milk, colostrums**	*E. faecalis* F1 and *W. confuse *F8 strains among 33 isolates	*In vitro* gastrointestinal conditions resistance, antimicrobial and antibiotic activity,	[39]
		*Lb. plantarum *WLPL04	*In vitro* gastrointestinal conditions resistance, antimicrobial and antibiotic activity, antiadhesion of pathogens, protection from harmful effect of sodium dodecyl sulfate, and anti-inflammatory properties	[40]
		9 of *Lb. gasseri, Bifidobacterium breve*, and *S. salivarius *strains	*In vitro* gastrointestinal conditions resistance, antimicrobial and antibiotic activity, agglutination properties	[41]
		*B. animalis *subsp. *lactis (B. lactis*) INL1	*in vivo* anti-inflammatory capacities	[42]

Animals	**Gastrointestinal tract**			
	(i) calves	*Lb. fermentum* V3B-08, *Weissella hellenica* V1V-30, *Lb. farciminis* B4F-06	*In vitro *gastrointestinal conditions resistance, antibiotic and antimicrobial susceptibility *In vivo* mice intestine colonization, immunomodulation	[43]
	(ii) pigs	3 of *Lb. salivarius* strains	*In vitro* antimicrobial activity	[44]
	(iii) goats	3 of *Pediococcus pentosaceus* LJR1, LJR5, and LJR9 strains	*In vitro *gastrointestinal conditions resistance, antibacterial activity, adhesion to the HCT-15 cells, anti-inflammatory properties	[45]
	(iv) fishes	15 of *Candida* sp., *R. mucilaginosa, Y. lipolytica, M. viticola, C. laurentii, D. hansenii*, and *S. cerevisiae* yeast strains	*In vivo* reduction of mortality associated to *V. anguillarum* challenge in zebrafish	[46]
	(v) bees	*Lb. johnsonii* CRL1647	*In vitro* antibacterial activity, high auto-agregation properties *In vivo* stimulate of bee egg-laying and vitality	[47, 48]

Fermented food	**Milk and dairy products**			
	(i) camel's milk	*L.* lactis KX881768, *Lb.plantarum* KX881772, *L. lactis *KX881782 and *Lb. plantarum* KX881779	*In vitro g*astrointestinal conditions resistance, antibiotic and antimicrobial susceptibility, auto- and co-agregation properties, cholesterol removal	[49]
	(ii) yak milk	*Lb. plantarum* YD5S and YD9S, *Lb. pentosus* YD8S, *Lb. paraplantarum* YD11S, *E. lactis* YHC20 and *E. faecium* YY1	*In vitro*: hypocholesteromic effect, acid tolerance, bile tolerance, bile salt hydrolase (BSH) activity, cell surface hydrophobicity	[50]
	(iii) goat's milk	*Lb. plantarum* and *Pediococcus acidilactici*	*In vitro g*astrointestinal conditions resistance, antibiotic and antimicrobial susceptibility, adhesion properties	[51]
	(iv) cow's milk	*Lb. helveticus* KII13 and KHI1 strains	*In vitro g*astrointestinal conditions resistance, adherence to Caco-2 cells, antimicrobial and cholesterol-lowering activity *In vivo* cholesterol-lowering activity in mice model	[52]
	(v) whey	16 of *Lb. plantarum* and *Lb. fermentum *strains	*In vitro *antibacterial activity, safety assessment	[53]
	(vi) traditional Greek dairy products	*S. thermophilus* ACA-DC 26 2 of *Lb. plantarum* ACA-DC 2640 and ACA-DC 4039 strains, *Lb. plantarum* ACA-DC 2640 and S. thermophilus ACA-DC 26 and ACA-DC 170	*In vitro *antibacterial activity, high adherence ability, anti-inflammatory properties	[54]
	(vii) traditional Polish cheeses	29 of *Lb. plantarum* strains	*In vitro *antibacterial activity	[55]
	(viii) Tibetan kefir grain	*Lb. kefiranofaciens* XL10	*In vitro *gastrointestinal conditions resistance, auto-aggregation properties *In vivo* modulation of gut microbiota, adhere and colonize to intestine tissue of mice	[56]
	(ix) Iranian Spar	*Lb. brevis* LSe	*In vitro* gastrointestinal conditions resistance, adhesion to Caco-2 cells, antioxidant and high selenium-tolerant activity	[57]
	**Raw fermented meat products**			
	(i) Thai fermented pork sausage	*Lb. plantarum* subsp. *plantarum *SKI19	*In vitro* gastrointestinal conditions resistance, adhesion to xylene and chloroform, antimicrobial activity, safety assessment	[58]
	(ii) *Harbin* dry sausages	*Pediococcus pentosaceus* R1, *Lb. brevis *R4, *Lb. curvatus *R5, and *Lb. fermentum*	*In vitro* gastrointestinal conditions resistance, auto-aggregation, adhesion to Caco-2 cells, antioxidant activity	[59]
	(iii) raw fermented Polish meat products	21 of* Lb. plantarum, Lb. brevis, Pd. pentosaceus* strains	*In vitro* gastrointestinal conditions resistance, antimicrobial activity, safety assessment	[60]
	(iv) cooked meat products	*E. *faecium UAM1	*In vitro* gastrointestinal conditions resistance, auto- and co- aggregation, adhesion to Caco-2 cells,	[61]
	**Fishes and seafood**			
	(i) *Hentak*, a fermented fish product of North-East India	*Lb. brevis* LAP2	*In vitro* gastrointestinal conditions resistance, auto-aggregation, hydrophobicity, antioxidant and antimicrobial potential	[62]
	(ii) Japanese fermented fish (funa-sushi)	heat-killed *Lb. paracasei* NFRI 7415	*In vivo* inhibition of mesangial proliferative glomerulonephritis by alcohol intake with stress in mice model	[63]
	(iii) Korean salted fermented seafood (Jeotgal)	*Lb. plantarum* JBCC105645 and JBCC105683	*In vitro* stimulation macrophages to produce IL-12 *In vivo* immunostimulation, inhibition of atopic dermatitis -like skin lesions and reduction serum IgE levels in mice model	[64]
	(iv) marine oyster	*E. faecium* HL7	*In vitro* antimicrobial activity, resistant to environmental stressors, antibiotic sensitivity	[65]
	**Pickled vegetables**			
	(i) kimchi	*Lactococcus lactis* KC24	*In vitro* gastrointestinal conditions resistance, antimicrobial properties, adhesion to Caco-2 cells, antioxidant, anti-inflammatory, anticancer activity,	[66]
	(ii) Polish fermented cabbage and cucumber	14 of *Lactobacillus* spp.	*In vitro* gastrointestinal conditions resistance, antimicrobial properties, adhesion to xylene, safety assessment	[67]
	(iii) cocoa fermentation	*Lb. fermentum* TcUESC01 and *Lb. plantarum* TcUESC02	*In vitro* antimicrobial properties, *In vivo* anti-inflammatory and immunomodulation activity	[68]
	(iv) Mexican alcoholic, non-distilled, fermented beverage (*Pulque)*	*Leuconostoc mesenteroides* strain P45	*In vitro* gastrointestinal conditions resistance, antimicrobial properties, *In vivo* anti-infective activity against *S. enterica* serovar Typhimurium in challenged mice	[69]
	(v) Korean fermented soybean paste	*P. acidilactici *SDL 1402, SDL 1405, SDL 1406, *Weissella *cibaria SCCB 2306, *S. * t*hermophilus* SCML 337, SCML 300 and *E*. *faecium* SC 54	*In vitro* gastrointestinal conditions resistance, auto- and co- aggregation ability, adhesion to xylene, safety assessment *In vivo* colonization ability and strongly attachment to *Caenorhabditis elegans* gut	[70]
	**Sourdough, cereal products**			
	(i) India fermented pearl millet porridge (*Kambu koozh*)	*Lb. fermentum* CFR5, CFR1, CFR4 and CFR2 and *Lb. delbrueckii *CFR6	*In vitro g*astrointestinal conditions resistance, bile salt hydrolase activity, auto-aggregation ability, antimicrobial and antioxidant activity, safety assessment	[71]
	(ii) Altamura dough	*S. cerevisiae* 2 and S*. cerevisiae *4	*In vitro g*astrointestinal conditions resistance, hydrophobic ability, antimicrobial activity, safety assessment	[72]

Non-fermented food	**Fruits and vegetables**			
	(i) byproducts of fruit pulp processing	*Lb. brevis* 59, *Lb. pentosus *129, *Lb. paracasei *108, *Lb. plantarum *49, and *Lb. fermentum *111	*In vitro g*astrointestinal conditions resistance, antimicrobial activity, safety assessment	[73]
	(ii) pineapple and pineapple peels	50 isolates of *Candida lusitaniae* and *Meyerozyma caribbica*	*In vitro g*astrointestinal conditions resistance, antimicrobial activity, safety assessment	[74]
	(iii) raw fruits and vegetables	48 of* Lactobacillus, Weissella *and *Pediococcus *strains	*In vitro g*astrointestinal conditions resistance, adhesion to Caco-2 cells, immunomodulatory properties, antimicrobial activity	[75]
	(iv) carrot	*Enterococcus durans* QU 49	*In vitro* bacteriocin production	[76]

Environment	**Food wastes**			
	(i) poultry slaughterhouse waste	*Lb. plantarum *LPL9, *Lb. ramnosus* LRH25, and *Lb. fermentum* LFE26	*In vitro g*astrointestinal conditions resistance, antimicrobial activity, adhesion to hydrocarbons	[77]
	(ii) moldy corn	*Bacillus amyloliquefaciens*	*In vitro* Zearalenone removal ability, *g*astrointestinal conditions resistance, antimicrobial activity	[78]
	**Air** in working and storage room of bakery	Strains of* Lb. plantarum* and *Lb. sanfranciscensis*	16S rRNA gene sequencing and amplified fragment length polymorphism analysis	[79]
	**Soils** of North East Himalayas	*Bacillus amyloliquefaciens* JF836079	*In vitro g*astrointestinal conditions resistance, adhesion to Caco-2 cells *In vivo* beneficial effect on Inflammatory Bowel Disease in mice	[80]
